# The Netrin-related domain of Sfrp1 interacts with Wnt ligands and antagonizes their activity in the anterior neural plate

**DOI:** 10.1186/1749-8104-3-19

**Published:** 2008-08-20

**Authors:** Javier Lopez-Rios, Pilar Esteve, Jose Maria Ruiz, Paola Bovolenta

**Affiliations:** 1 Departamento de Neurobiología Molecular Celular y del Desarrollo, Instituto Cajal, CSIC, Dr. Arce 37, Madrid, 28002, Spain; 2 Developmental Genetics, DBM Centre for Biomedicine, University of Basel, Mattenstrasse, CH-4058, Basel, Switzerland; 3 CIBER de Enfermedades Raras (CIBERER), Dr. Arce 37, Madrid, 28002, Spain

## Abstract

**Background:**

Secreted frizzled related proteins (SFRPs) are multifunctional modulators of Wnt and BMP (Bone Morphogenetic Protein) signalling necessary for the development of most organs and the homeostasis of different adult tissues. SFRPs fold in two independent domains: the cysteine rich domain (Sfrp_CRD_) related to the extracellular portion of Frizzled (Fz, Wnt receptors) and the Netrin module (Sfrp_NTR_) defined by homologies with molecules such as Netrin-1, inhibitors of metalloproteinases and complement proteins. Due to its structural relationship with Fz, it is believed that Sfrp_CRD _interferes with Wnt signalling by binding and sequestering the ligand. In contrast, the functional relevance of the Sfrp_NTR _has been barely addressed.

**Results:**

Here, we combine biochemical studies, mutational analysis and functional assays in cell culture and medaka-fish embryos to show that the Sfrp1_NTR _mimics the function of the entire molecule, binds to Wnt8 and antagonizes Wnt canonical signalling. This activity requires intact tertiary structure and is shared by the distantly related Netrin-1_NTR_. In contrast, the Sfrp1_CRD _cannot mirror the function of the entire molecule *in vivo *but interacts with Fz receptors and antagonizes Wnt8-mediated β-catenin transcriptional activity.

**Conclusion:**

On the basis of these results, we propose that SFRP modulation of Wnt signalling may involve multiple and differential interactions among Wnt, Fz and SFRPs.

## Background

Secreted frizzled related proteins (SFRPs) compose a family of soluble factors widely involved in the control of embryonic development and the homeostasis of adult tissues. Members of this family were independently isolated using a variety of approaches and immediately proposed as Wnt signalling inhibitors because of their ability to interfere with Wnt-induced embryonic axis duplication and forebrain development in *Xenopus *[1,2]. Many studies have thereafter confirmed that addition of SFRPs can block Wnt-mediated signalling activation in different experimental paradigms showing possible binding preferences between SFRP and Wnt pairs (reviewed in [3]). Whether SFRP-mediated interference with Wnt signalling activation is the result of a single biochemical interaction between Wnt and SFRPs or instead reflects multiple binding mechanisms among SFRP, Wnt and their Frizzled (Fz) receptors is, however, a still unresolved issue.

Indeed, SFRP molecules fold in two independent domains: an amino-terminal cysteine-rich domain (CRD) and a carboxy-terminal Netrin-related motif (NTR) [4,5]. The Sfrp_CRD _contains ten cysteines with a pattern of five disulfide bridges identical to that of the extracellular CRD of Fz [6,7]. Due to this structural relationship, it is generally assumed that Sfrp-mediated Wnt signalling inhibition results from the interaction between the ligand and Sfrp_CRD_, which has been actually shown to immunoprecipitate with Wnt1 and Wnt2 [8,9]. However, Sfrp_CRD _can also form homo- and heterodimers with the CRD domain of Fz receptors [8,10], suggesting potential alternative mechanisms of action.

The carboxy-terminal Sfrp_NTR _is separated from the Sfrp_CRD _by a linker region and is characterized by the presence of several conserved blocks of hydrophobic residues and a pattern of six conserved cysteines. NTR domains with similar features are found in a wide range of otherwise unrelated proteins, including Netrin-1, tissue inhibitors of metallo-proteinases (TIMPs), complement proteins and type I procollagen C-proteinase enhancer proteins (PCOLCEs) [11]. Despite an initial suggestion that the Sfrp_NTR _may interact with Wnt ligands [4], the participation of this domain in SFRP function has not been addressed.

Here, we have combined biochemical studies, mutational analysis and functional assays in cell culture and medaka-fish embryos to test the functional relevance of the Sfrp_NTR _in Wnt signalling modulation. We show that the Sfrp1_NTR _mimics the function of the full-length Sfrp1, binds to Wnt ligands and prevents Wnt canonical signalling activation, effects shared by distantly related NTR domains such as that of Netrin-1. In contrast, Sfrp1_CRD _fails to interact with Wnt but binds to Fz receptors, possibly explaining the potential that the CRD has to inhibit Wnt signalling. We thus conclude that SFRPs modulate Wnt signalling by interacting with both Wnt ligands and Fz receptors but through different domains of the molecule and propose possible models of SFRP function that may reconcile data available in the literature.

## Results

### Sfrp1_NTR _mimics the effect of the full-length protein in the anterior neural plate

**Figure 1:**
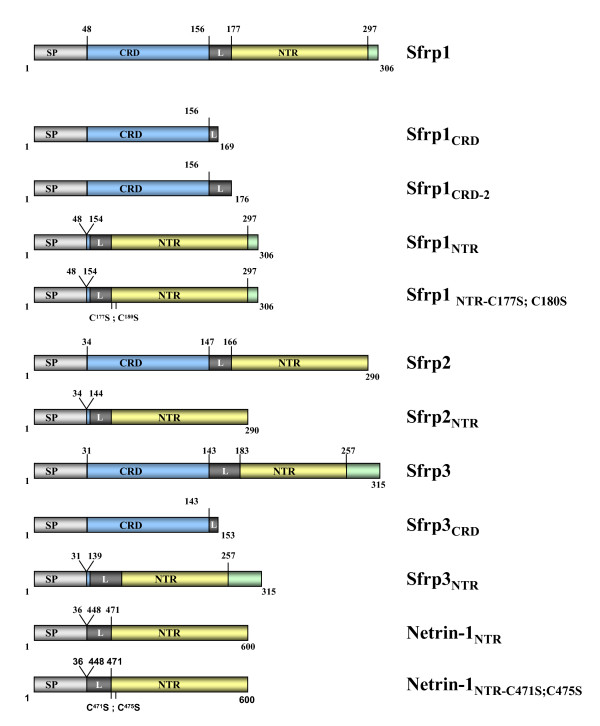
**Schematic representation of the different constructs used in this study**. Construct organization and generated mutations are indicated in the drawings. Light grey boxes, signal peptide (SP); light blue boxes cysteine e-rich domain (CRD); dark grey boxes, linker (L); yellow boxes, Netrin-related domain (NTR); green boxes, carboxy-terminal end of the protein.

**Figure 2:**
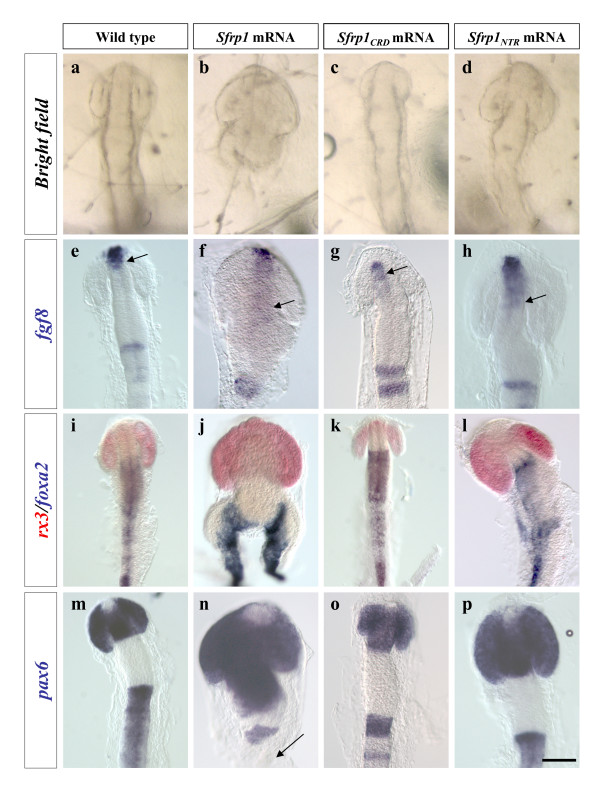
***Sfrp1***_***NTR***_, **but not *****Sfrp1***_*CRD*_,** mimics the phenotype induced by the over-expression of full-length *Sfrp1*.(a-p) **All the panels are dorsal views of embryos at stage 19–20 (optic vesicle stage) injected with *GFP *mRNA alone (control) (a,e,i,m) or together with *olSfrp1 *(b,f,j,n), *Sfrp1*_*CRD *_(c,g,k,o) or *Sfrp1*_*NTR *_(d,h,l,p) mRNA. Embryos in (i-l) have been processed for double *in situ *hybridization with *rx3 *(red) and *foxA2 *(blue) probes. All other embryos were hybridized for one probe as indicated. Note how anterior markers are dramatically expanded in both the *Sfrp1 *and *Sfrp1*_*NTR *_injected embryos (arrow in e-h), while over-expression of *Sfrp1*_*CRD *_leads to a reduction of forebrain structures. *Sfrp1 *injected embryos also display axial duplications (j) and posterior truncations (b,j, arrow in n). See Table 1 for details. Scale bar: 0.1 mm.

**Figure 3:**
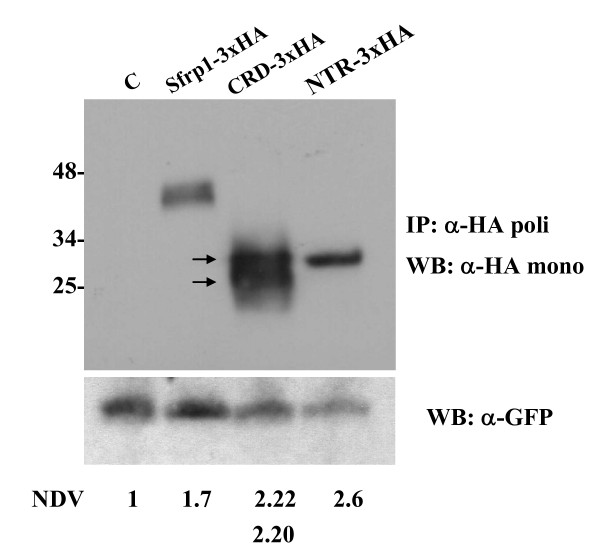
***Sfrp1***, ***Sfrp1***_***CRD ***_**and *****Sfrp1***_***NTR***_**mRNAs are translated at comparable levels when overexpressed *in vivo***. Western blot (WB) analysis of lysates from embryos injected with equimolecular amounts of *Sfrp1-3xHA*, *Sfrp1*_*CRD *_-*3xHA *or *Sfrp1*_*NTR*-_*3xHA *mRNAs together with *GFP *mRNA as a tracer. Embryos were collected at St26 and their lysates were precipitated with a polyclonal anti-HA and blotted with monoclonal anti-HA. To account for possible variations in the amount of injected mRNA, the expression levels of Sfrp peptides were normalized against those of the co-injected EGFP protein. Note that the normalised density values of the three peptides (NDV) are very similar. Note also that Sfrp_CRD _runs as a doublet that may represent monomeric and dimeric forms (arrows) or post-translational modifications. IP, immunoprecipitation.

*Sfrp1 *is expressed in the anterior neural plate and is required to establish the prospective eye territory [12,13]. In line with this idea, *Sfrp1 *(Figure 1) over-expression in the medaka fish leads to a morphologically evident enlargement of the forebrain, posterior truncations and axial duplications (Figure 2b; Table 1). These defects correlate with the expansion of the expression domains of telencephalic, optic vesicle and diencephalic markers such as *fgf8*, *rx3 *and *pax6 *(Figure 2f,J,n), the alteration of the axial mesoderm marker *foxa2 *(Figure 2j) and the loss of the posterior domain of *pax6 *(arrow in Figure 2n). To determine whether the NTR domain of Sfrp1 contributed to this effect, we generated expression constructs encoding truncated (Sfrp1_CRD_) or chimerical peptides (Sfrp1_NTR_; harbouring its own signal peptide to ensure proper secretion; Figure 1) that comprised the two independent domains in which the protein has been shown to fold [5]. Notably, injections of equimolar concentrations of *Sfrp1*_*NTR *_mRNA led to the enlargement of the forebrain and the expansion of anterior markers (Table 1; Figure 2d,h,l,p), as observed after the over-expression of full-length *Sfrp1*. Although all peptides seemed to be produced at comparable levels (Figure 3; see below), higher concentrations of *Sfrp1*_*NTR *_mRNA were necessary to induce posterior truncations or axial duplications (data not shown), suggesting a differential requirement of Sfrp1_NTR _along the antero-posterior axis. Alternatively, the peptide was less effective than the entire Sfrp1 protein, perhaps due to a difference in maturation and half-life or diffusion range. Another possible explanation is that monomeric Sfrp1_NTR _is less effective than the full-length protein, since protein dimerization through the CRD motif has been previously described [8,10].

**Table 1:** Anteriorised phenotypes induced by over-expression of different *Sfrp *variants

Injected mRNA	Percentage of embryos showing an enlarged forebrain
*Sfrp1 *(200 ng/μl; n = 70)	91
*Sfrp1*_*CRD *_(100 ng/μl; n = 162)	0 (55)*
*Sfrp1*_*CRD*-2 _(100 ng/μl; n = 86)	0 (48)*
*Sfrp1*_*NTR *_(120 ng/μl; n = 158)	65
*Sfrp1*_*NTR*-*C*177*S*;*C*180*S *_(120 ng/μl; n = 48)	13
	
*Sfrp2 *(200 ng/μl; n = 62)	93
*Sfrp2*_*NTR *_(120 ng/μl; n = 47)	47
	
*Sfrp3 *(200 ng/μl; n = 51)	4 (42; n = 40)^†^
*Sfrp3*_*NTR *_(120 ng/μl; n = 38)	3 (27; n = 56)^†^
*Sfrp3*_*CRD *_(100 ng/μl; n = 36)	0 (0; n = 75)^†^
	
*Netrin-1*_*NTR *_(120 ng/μl; n = 61)	56
*Netrin-1*_*NTR*-*C*471*S*;*C*475*S *_(120 ng/μl; n = 40)	42

Percentage of embryos showing an anteriorised phenotype upon injection of equimolecular amounts of mRNAs encoding different variants of Sfrp or Netrin-1 proteins, as shown in Figures 1 and 3 and Additional file 2. The anteriorised phenotype was scored by an evident morphological expansion of the prosencephalic tissue at late neurula stages. The percentage in brackets marked with asterisks represent the frequency of embryos showing a reduction in the size of the forebrain (instead of an increase; see text for details). The percentages in brackets marked with a dagger represent the frequency of appearance of the phenotype at higher concentration: 500 ng/μl for *Sfrp3 *and 300 ng/μl for *Sfrp3*_*NTR *_and *Sfrp3*_*CRD*_.

Quite surprisingly, over-expression of Sfrp1_CRD_, the domain postulated to mediate SFRP-Wnt interactions, did not result in comparable phenotypes (Table 1). Instead, *Sfrp1*_*CRD *_mRNA injected embryos presented a small but appreciable reduction of the forebrain (Figure 2c), which was associated with a diminished expression of prosencephalic markers (Figure 2g,k,o). Forebrain reduction was more evident at earlier stages of differentiation even with lower doses of mRNA (data not shown), supporting that the Sfrp1_CRD _gain-of-function phenotype did not reflect lower levels of peptide expression. Accordingly, Western blot analysis of embryos injected with haemagglutinin (HA)-tagged versions of the peptides indicated that *Sfrp1 *and *Sfrp1*_*NTR *_mRNA were efficiently translated at comparable levels while the *Sfrp1*_*CRD *_mRNA was produced in a larger amount, which existed in a monomeric and possibly a dimeric form (Figure 3).

**Figure 4:**
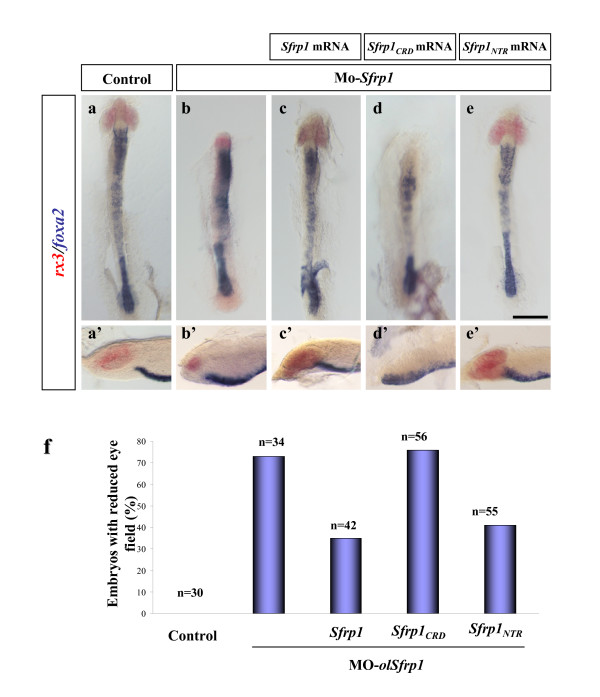
***Sfrp1***_***NTR ***_**but not*****Sfrp1***_***CRD***_, **rescues the phenotype induced by knocking-down *Sfrp1***. **(a-e') **All the panels are dorsal (a-e) and lateral (a'-e') views of embryos at stage 19–20 injected with *GFP *mRNA alone (a,a'), *Mo-olSfrp1 *alone (b,b') or co-injected with *Sfrp1 *(c,c'), *Sfrp1*_*CRD *_(d,d') or *Sfrp1*_*NTR *_(e,e') mRNAs as indicated in the panels. Embryos were hybridised for *rx3 *(eye field) and *foxA2 *(axial mesoderm) both visualised in blue. Optic vesicles fail to develop in embryos injected with Mo-*Sfrp1*, as judged by the reduction in *rx3 *expression (b,b'). This defect is reverted by the co-injection of *Sfrp1 *and *Sfrp1*_*NTR *_mRNAs in 50% of the embryos (c,c',e,e',f) but not by that of *Sfrp1*_*CRD *_(d,d',f) mRNA, where the reduction of the eye field is even more pronounced than that observed with the Mo-*Sfrp1 *alone. Note that *Sfrp1 *mRNA not only rescues the effect of Mo-*Sfrp1 *but also induces a partial over-expression phenotype (compare (c,c') with Figure 2a,j). **(f) **Quantification of the rescue efficiency in the different conditions. Scale bar: 0.2 mm.

Morpholino (Mo)-based knock-down of *Sfrp1 *expression results in embryos with a reduced eye field associated, in the most affected embryos, with a shortening and widening of the antero-posterior axis [13] (compare Figure 4b,b' with Figure 4a,a'). Low concentrations of *Sfrp1 *mRNA are sufficient to completely rescue this phenotype in a large part of the embryos [13] (Figure 4c,c',f). If *Sfrp1*_*NTR *_can mimic the effect of the entire molecule, it should also be able to rescue the effects of Mo interference. Supporting this hypothesis, co-injection of Mo-*Sfrp1 *and *Sfrp1*_*NTR *_mRNA rescued the size of the eye field of the treated embryos with efficiency similar to that of *Sfrp1 *(Figure 4e,e',f). In contrast, *Sfrp1*_*CRD *_mRNA did not counteract the Mo-*Sfrp1 *induced phenotype (Figure 4d,d',f) and even appeared to exacerbate it, in line with the over-expression studies.

Together, these data suggested that the molecular events induced by the two domains of Sfrp1 were probably different in nature. The *Sfrp1*_*CRD*_-induced phenotype was difficult to explain according to the generally accepted view that this domain binds Wnt ligands and antagonizes their activity. In contrast, the strong anteriorisation observed after *Sfrp1 *and *Sfrp1*_*NTR *_over-expression could be easily explained as the result of an early and generalized antagonism of the canonical Wnt pathway, since inhibition of this pathway induces similar anteriorised and dorsalised phenotypes in both fish and *Xenopus *embryos [1,2,13].

**Figure 5:**
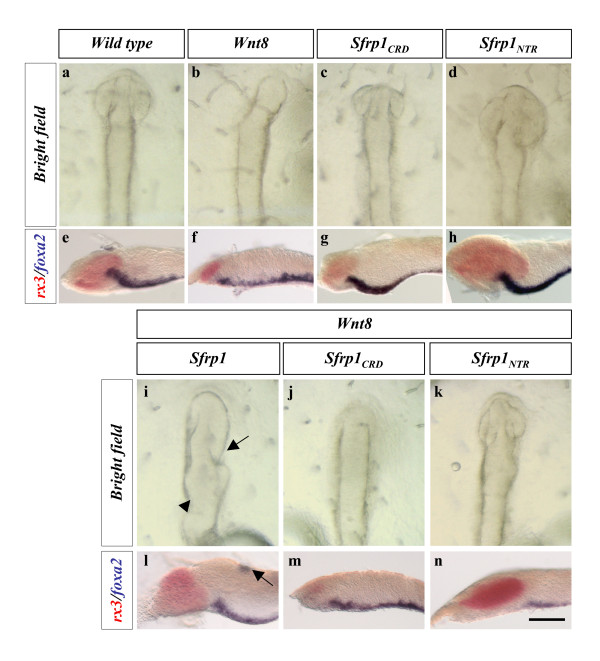
***Sfrp1***_***NTR***_**rescues the phenotype induced by *Wnt8 *over-expression**. All the panels are dorsal **(a-d; i-k) **or lateral **(e-h; l-n) **views of embryos at stage 19–20 injected with *GFP *mRNA (a,e); *GFP *together with *Wnt8 *(b,f), *Sfrp1*_*CRD *_(c,g), *Sfrp1*_*NTR *_(d,h) or *Wnt8 *together with *Sfrp1 *(i,l), *Sfrp1*_*CRD *_(j,m) or *Sfrp1*_*NTR *_(k,n) mRNA as indicated. Optic vesicles fail to develop in embryos injected with *Wnt8 *mRNA, as judged by the reduction in *rx3 *expression (b,f). (i-n) This defect is reverted by *Sfrp1 *(i,l) and *Sfrp1*_*NTR *_(k,n) but not by *Sfrp1*_*CRD *_(j,m) co-expression. Note that *Wnt8*-induced forebrain reduction is somewhat enhanced in the presence of *Sfrp1*_*CRD*_. Embryos were processed for double *in situ *hybridization with *rx3 *(red) and *foxA2 *(blue) probes. Arrows and arrowheads (i,l) indicate moderate expansion of anterior tissue and axial duplications induced by *Sfrp1 *over-expression. See Tables 1 and 2 for details. Scale bar: 0.18 mm (a-d,i-k); 0.25 mm (e-h;l-m).

To investigate this possibility, we next assayed whether injection of *Sfrp1 *and *Sfrp1*_*NTR *_could alleviate the phenotypes caused by *Wnt8*-mediated activation of canonical Wnt signalling. As previously shown in other species [14,15], *Wnt8 *over-expression in medaka fish embryos led to a strong reduction of the forebrain associated with loss of the *rx3*-positive optic vesicles (Table 2; compare Figure 5a,e with Figure 5b,f). These anterior defects were similar to those observed after *Sfrp1*_*CRD *_injections (Figures 2c,k and 5c,g; Table 2) but opposite to those induced by *Sfrp1 *or *Sfrp1*_*NTR *_over-expression (Figures 2b,d,j,l and 5d,h; Table 2). Upon co-injection, *Wnt8 *and *Sfrp1 *mRNAs appeared to counteract each other's activity, resulting in mildly anteriorised embryos (Figure 5i,l; Table 2) that, however, still presented partial posterior truncations or axis duplications (Figure 5i,l). This suggests that, in the concentration range tested, *Wnt8 *cannot completely counteract *Sfrp1*-induced axial defects. In agreement with our previous observations, *Sfrp1*_*NTR *_mRNA abrogated the *Wn8*-induced phenotype, restoring almost completely the size of the *rx3 *expression domain (Figure 5k,n; Table 2; compare to control embryos in Figure 5a,b). In contrast, *Sfrp1*_*CRD*_, rather than counteracting, accentuated the reduction of the forebrain induced by *Wnt8 *(Figure 5j,m).

**Table 2:** Antagonistic interaction between *Sfrp *variants and *Wnt8/Wnt5*

	*Wnt8 *(50 ng/μl)		*Wnt5 *(50 ng/μl)	
		
Co-injected mRNA	n	Percentage of embryos showing a reduced forebrain	n	Percentage of embryos showing a reduced forebrain
None (*Wnt8*/*Wnt5 *alone)	107	88	81	86
*Sfrp1 *(200 ng/μl)	81	0 (30)	78	0 (90)
*Sfrp1*_*CRD *_(100 ng/μl)	68	96	72	83
*Sfrp1*_*NTR *_(120 ng/μl)	110	20 (14)	90	7 (60)
*Sfrp3 *(200 ng/μl)	96	87	98	69
*Sfrp3*_*CRD *_(120 ng/μl)	117	92	81	93
*Sfrp3*_*NTR *_(100 ng/μl)	94	60	89	72

Percentage of embryos showing a size reduction of the forebrain/optic vesicles upon injection of equimolecular amounts of mRNAs encoding *Wnt8 *or *Wnt5 *together with different variants of *Sfrp1 *and *Sfrp3 *mRNAs. Representative embryos are shown in Figure 2 and Additional file 2. *Wnt8*-induced forebrain reduction is much more severe (optic vesicles are completely absent), than that observed upon *wnt5 *over-expression, where the optic vesicles are, in general, significantly reduced in size but still visible. In the case of *Wnt *and *Sfrp1 *and *Sfrp1*_*NTR *_co-injections, the number shown in brackets represents the frequency of appearance of the anteriorised phenotype (enlarged forebrain tissue), which is reduced compared to the over-expression of the given *Sfrp *construct alone (Table 1).

Altogether, these results challenged the view that the CRD domain of the Sfrp1 protein plays an important role in Wnt antagonism. To exclude the possibility that inadequate folding or destabilization of the Sfrp1_CRD _construct could mislead this interpretation, we designed an additional construct encoding the CRD and the entire linker region (Sfrp1_CRD2_; Figure 1) to ensure proper folding of the Sfrp1 CRD domain [5]. Over-expression of this new construct, *Sfrp1*_*CRD*2_, caused phenotypes similar to those observed upon *Sfrp1*_*CRD *_injection (Additional file 1). As an alternative explanation, the behaviour of the Sfrp1_CRD _could reflect a peculiarity of this specific member of the SFRP family. Therefore, the CRD domain of Sfrp3 (Sfrp3_CRD_; Figure 1), the family member that diverges the most from *Sfrp1 *[13], was also analyzed. Interestingly, over-expression of *Sfrp3*_*CRD *_had no morphologically evident effects on embryonic development, even at high concentrations (Additional file 1; Table 1) and, in contrast to *Sfrp1*_*CRD*_, failed to enhance *Wnt8*-induced phenotype (Additional file 1; Table 2).

**Figure 6:**
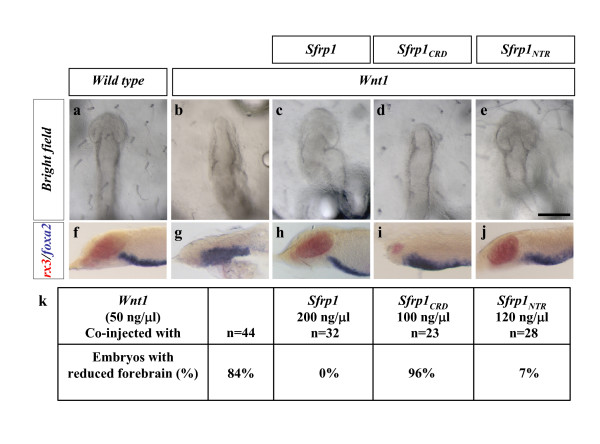
***Sfrp1***_***NTR***_**rescue ability is observed also with *Wnt1*, another canonical ligand**. **(a-e) **Dorsal views of embryos at stage 19–20 injected with *GFP *mRNA (a), *Wnt1 *(b), *or Wnt1 *together with *Sfrp1 *(c), *Sfrp1*_*CRD *_(d), or *Sfrp1*_*NTR *_(e) mRNAs. **(f-j) **Lateral views of embryos processed for double *in situ *hybridization with *rx3 *(red) and *foxA2 *(blue) probes injected with the same mRNAs, respectively. The phenotype induced by *Wnt1 *mRNA injection is very similar to that observed with *Wnt8*: the optic vesicles fail to develop (b), with a reduction in *rx3 *expression (g). This defect is reverted by *Sfrp1 *(c,h) and *Sfrp1*_*NTR *_(e,j) but not by *Sfrp1*_*CRD *_(d,i) co-expression. **(k) **Percentage of embryos showing reduction in the size of the forebrain/optic vesicles upon injection of *Wnt1 *mRNA or together with equimolecular amounts of mRNAs encoding different variants of *Sfrp1*. Scale bar: 0.2 mm.

As a third possibility, we considered that our results could reflect differential affinities between SFRPs and this particular Wnt ligand [16]. Therefore, co-injection studies were repeated using two different Wnts: Wnt1, another canonical Wnt that, like Wnt8, can induce posteriorisation of the embryos [17], and Wnt5, which is thought to activate preferentially the non-canonical Wnt signalling pathway [18]. As shown in Figure 6, injections of *Sfrp1 *and *Sfrp1*_*NTR *_counteracted the phenotype caused by Wnt1-induced phenotype with efficiencies that were very comparable to those observed with Wnt8, while *Sfrp1*_*CRD *_did not. *Wnt5 *over-expression in fish and *Xenopus *embryos leads to variable phenotypes [18,19], including defects in axial extension and reduction of the optic vesicle size, albeit less dramatic than those observed with *Wnt8 *(Additional file 1). Co-injection of Wnt5 with *Sfrp1*_*CRD *_or *Sfrp3*_*CRD *_did not rescue the *Wnt5*-induced phenotype (Additional file 1; Table 2), thus diminishing the relevance of the Sfrp_CRD _as a Wnt ligand antagonist. In contrast, our results suggest a relevant role of Sfrp1_NTR _in antagonizing Wnt activity.

### Sfrp1_NTR _effects are shared by distantly related NTRs and require intact tertiary structure

**Figure 7:**
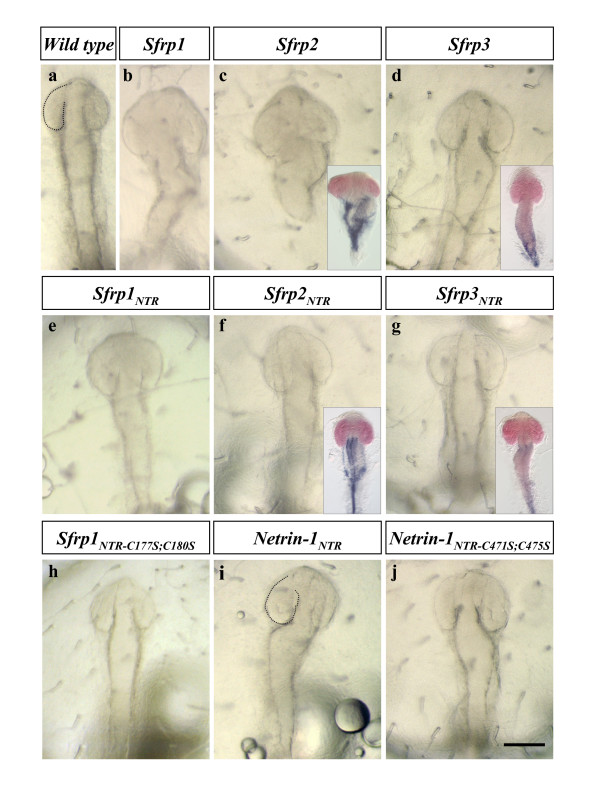
**Distantly related NTR domains mimic the activity of *Sfrp1*_*NTR *_with different efficiencies**. **(a-j) **Brightfield views of embryos injected with different full-length or chimerical mRNAs as indicated. Insets in (c,d,f,g) correspond to embryos processed for double *in situ *hybridization for *rx3 *(red) and *foxA2 *(blue). Note that injections of *Sfrp1 *(b), *Sfrp2 *(c, and inset) lead to similar expansion of anterior structures compared to control embryos (a), while *Sfrp3 *has a very weak anteriorizing effect (d) observed only in 4% of the injected embryos (Table 1; inset in (d) shows an embryo injected with a high dose (500 ng/μl) of *Sfrp3 *mRNA). Similarly, *Sfrp3*_*NTR *_induces a weak anteriorisation at a low frequency (embryo shown in (g); Table 1), whereas the distantly related NTR motif from Netrin-1 (i) induces an expansion of the forebrain as observed with Sfrp1_NTR_. Note that the functionality of the NTR domain depends on an intact tertiary structure, since cysteine to serine mutations in *Sfrp1*_*NTR*-*C*177*S*;*C*180*S *_and *Netrin-1*_*NTR*-*C*471*S*;*C*475*S *_constructs (h,j) induce a total or partial loss of the effect. See Table 1. Scale bar: 0.1 mm.

To explore this possibility further, we next investigated whether the relevance of the NTR domain in antagonizing Wnt ligands could be extended to other SFRP family members or even to distantly related NTR domains [11]. According to phylogenetic analysis, the SFRP family is composed of three subfamilies: *Sfrp1/2/5*, *Tlc/Sizzled *and the very divergent *Sfrp3/4 *[13]. We thus compared the activity of *Sfrp1 *and *Sfrp1*_*NTR *_with equivalent constructs from *Sfrp2 *and *Sfrp3 *(Figure 1), close and a divergent members of the SFRP family, respectively. Furthermore, we also chose to analyze the NTR domain of Netrin-1 (Figure 1), a secreted protein involved in axon guidance where the NTR domain was first identified [11,20] as a distantly related module. When assayed for their ability to reproduce the *Sfrp1 *over-expression phenotype (Figures 2b and 7b), *Sfrp2 *and *Sfrp2*_*NTR *_displayed a significant anteriorising activity almost identical to that of *Sfrp1 *and *Sfrp1*_*NTR*_, respectively (Figure 7c,f; Table 1), while *Sfrp3 *and *Sfrp3*_*NTR *_had a much weaker activity and expansion of anterior markers was only observed upon injection of high mRNA concentrations (Figure 7d(inset),g; Table 1). Intriguingly, *Netrin-1*_*NTR *_mRNA injections led to a mild expansion of the forebrain at lower frequency than those of *Sfrp1*_*NTR *_(Figure 7i; Table 1). These results indicate that, despite the evolutionary distance, this module can mimic SFRP function, presumably by binding to endogenous Wnt.

We next asked whether the tertiary structure of Sfrp1_NTR _was important for its function. The NTR motif is, in general, poorly conserved and mainly defined by the presence of six conserved cysteine residues that form three disulfide bonds [5,11]. Mutations of the first two of these residues (Cys177 and Cys180) are predicted to disrupt two disulfide bonds, thus destabilizing the tertiary structure of the NTR domain. Indeed, over-expression of such a mutated construct (Sfrp1_NTR-C177S;C180S_; Figure 1) did not alter medaka embryonic development (Figure 7h; Table 1), indicating that intact tertiary structure of the NTR motif is required for Sfrp1 activity. Notably, analogous mutations of the first two conserved cysteines of the Netrin-1_NTR _(Netrin-1_NTR-C471S;C475S_; Figure 1) also interfered with, but surprisingly not totally abolished, the anteriorising activity of this domain (Figure 7j; Table 1).

Altogether, these data strongly support that the NTR domain has a relevant role in mediating SFRP function and that this role is conserved also in distantly related domains.

### Sfrp1_NTR _and Sfrp1_CRD _bind to Wnt8 and Frizzled, respectively, antagonizing canonical signalling

In agreement with our finding that NTR domains of SFRPs are functionally relevant to Wnt signalling modulation, *in vitro *studies of the interaction between Sfrp1 and Wingless have mapped the relevant SFRP binding site to the carboxyl terminus of the protein [4]. To assess whether a similar biochemical interaction between Wnt8 and Sfrp1_NTR _could explain our over-expression experiments in medaka fish embryos, we challenged Wnt8 interaction with the two Sfrp1 domains.

**Figure 8:**
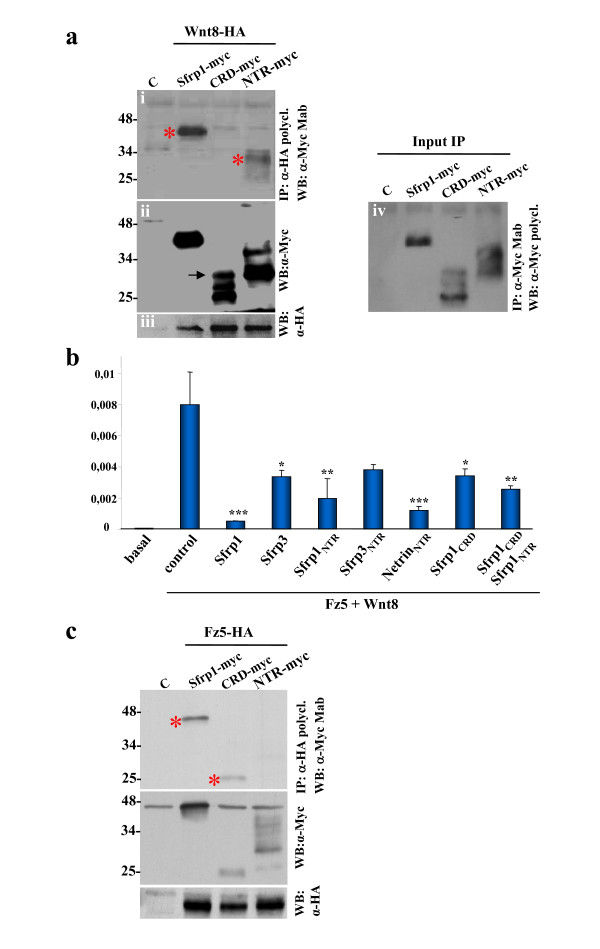
**Sfrp1_NTR _and Sfrp1_CRD _bind to Wnt8 and Frizzled-5, respectively, antagonizing canonical signalling**. **(a) **HEK 293T cells were transiently transfected with Wnt8-HA, Sfrp1-myc, Sfrp1_CRD-myc _or Sfrp1_NTR-myc _expression constructs. Conditioned media containing similar amount of each of the Sfrp1-myc derivates (ii) were mixed with conditioned media from Wnt8-HA (iii) or from mock transfected cells (Additional file 2). Proteins from mixed conditioned media were precipitated with a polyclonal anti-HA and blotted with a monoclonal anti-myc (i). In these conditions, both Sfrp1-myc and Sfrp1_NTR-myc _(red asterisks) specifically co-immunoprecipitated with Wnt8-HA, while Sfrp1_CRD-myc _did not. Comparable levels of Sfrp1 and its derivatives were immunoprecipitated (iv). Note that Sfrp1_NTR-myc _migrates as a smear due to post-translational glycosylation. Sfrp1_CRD-myc _likely suffers similar post-translational modifications and possibly forms dimers (arrow in (ii)) that do not completely dissociate. **(b) **Cells dissociated from E5 embryonic retinas were co-transfected with a reporter plasmid containing 4 × Lef-1 responsive element together with *Wnt8*, *Fz5 (*100 ng) in combination with the PCDNA plasmid alone (200 ng) or containing *Sfrp1*, *Sfrp3*, *Sfrp1*_NTR_, *Sfrp3*_NTR_, *Netrin-1*_NTR _or *Sfrp1*_*CRD *_constructs as indicated in the graph. *Wnt8*/*Fz5 *co-transfection activated the reporter expression 140-fold. This activation was strongly inhibited by the addition of Sfrp1, Netrin-1_NTR_, Sfrp1_NTR _or the combination of Sfrp1_NTR _and Sfrp1_CRD_. Equivalent amounts of Sfrp3, Sfrp3_NTR _or Sfrp1_CRD _alone were less effective. Data represent means ± standard error from three separate experiments performed in triplicates (*p < 0.05; **p < 0.01; ***p < 0.001; Student's *t*-test). **(c) **HEK 293T cells were transiently co-transfected with plasmids encoding *Sfrp1-myc*, *Sfrp1*_*CRD*-*myc *_or *Sfrp1*_*NTR*-*myc *_together with *Fz5-HA *expression vector (a) or PCDNA vector (Additional file 2). Proteins from cell lysates were precipitated with anti-HA and then blotted with anti-myc antibody. Note that Sfrp1 and Sfrp1_CRD _(red asterisks) interacted with Fz5 while the Sfrp1_NTR _did not. IP, immunoprecipitation; WB western blot.

To mimic the physiological conditions of the extracellular interaction between Wnt and SFRPs, we collected conditioned media derived from HEK 293T cells separately transfected with *Wnt8-HA*, *Sfrp1-myc*, *Sfrp1*_*NTR*-*myc *_or *Sfrp1*_*CRD*-*myc*_. The levels of proteins present in the conditioned media were carefully evaluated and equivalent amounts of Wnt8 (Figure 8a(iii)) were incubated with comparable quantities of either Sfrp1 or its derivatives (Figure 8a(ii)) and used for co-immunoprecipitation assays. Pull-downs with anti-HA IgG revealed that both Sfrp1-myc and Sfrp1_NTR-myc _specifically interacted with Wnt8-HA, while Sfrp1_CRD-myc _did not (Figure 8a(i)). Comparable levels of Sfrp1 and its derivatives were pulled down with anti-myc monoclonal antibodies (Figure 8a(iv)), minimising the possibility that the lack of Sfrp1_CRD_-Wnt interaction might be due to a less efficient immunoprecipitation of the Sfrp1_CRD_. Reverse pull-downs with a polyclonal anti-myc antiserum confirmed these results (Additional file 2).

To further test the functionality of this interaction in β-catenin-mediated Wnt signalling and to compare it with that of other NTR domains, we performed TCF-luciferase reporter-based assays in embryonic retinal cells, where β-catenin-mediated transcriptional activity is physiologically low [12]. We thus transfected retina cells with *Fz5*, a Wnt β-catenin associated receptor expressed in the anterior neural plate [21] to ensure Wnt8-mediated signalling activation [22]. Fz5 alone or in combination with Sfrp1, Sfrp1_CRD _or Sfrp1_NTR _did not modify basal β-catenin activity (Additional file 3). Instead, co-transfection or addition of Sfrp1, Sfrp1_NTR _or Netrin-1_NTR _conditioned media strongly inhibited reporter activity induced by Wnt8 and Fz5 over-expression (Figure 8b; Additional file 3). Equivalent amounts of Sfrp3 or Sfrp3_NTR _were less effective (Figure 8b), in good agreement with what is observed in medaka fish embryos (Figure 7). In apparent contrast with immunoprecipitation experiments, co-transfection of *Sfrp1*_*CRD *_also resulted in a significant decrease in reporter activity (Figure 8b). Notably, co-transfection with *Sizzled *or *Sizzled*_*CRD*_, a SFRP family member that does not appear to interfere with Wnt signalling [23], had a weaker activity (Additional file 3).

Sfrp1 has been shown to form complexes with Fz6 [8] and Fz2 [24], while crystallographic studies have shown that Fz8_CRD _and Sfrp3_CRD _can form dimers [10]. It was possible, therefore, that Sfrp1_CRD_-mediated inhibition of β-catenin transcriptional activity could result from Sfrp1_CRD _binding to the Fz5 receptor, thus preventing signal activation as previously proposed [8]. To test this possibility, we performed co-immunoprecipitation studies using cell lysates from HEK 293T cells transfected with *Fz5-HA*, *Sfrp1-myc *or its derivatives or co-transfected with *Fz2-HA*, as a positive control [24], and *Sfrp1-myc *or its derivatives. As shown in Figure 8c, both Sfrp1 and Sfrp1_CRD_, but not Sfrp1_NTR_, interacted with Fz5-HA, supporting the possibility that Sfrp1_CRD _could impede Fz5 activation in TCF-luciferase reporter-based assays by competing with Wnt8 for binding to the Fz receptor. A similar interaction was also observed between Fz2-HA and Sfrp1_CRD-myc _as well as with the entire protein (Additional file 2), confirming and extending previous studies [24].

## Discussion

Wnt signalling contributes to the regional specification of the anterior neural plate. Acquisition of diencephalic, eye and telecencephalic identities, however, require a differential contribution from canonical and non-canonical Wnt pathways, which are regulated by different Wnt antagonists, including *Sfrp1 *[25]. Accordingly, Mo-based knock-down of *Sfrp1*, a Wnt antagonist broadly expressed in the anterior neural plate, strongly reduces the eye field size, concomitantly expanding the telencephalic but not the diencephalic or mesencephalic territories in the medaka fish [13]. Conversely, *Sfrp1 *over-expression leads to expansion of the forebrain associated with posterior truncations and axial duplications [13]. Taking advantage of these activities, we have shown here that the NTR domain of Sfrp1 mimics the function of the full-length protein, binds to Wnt8 and antagonizes Wnt-canonical signalling. This activity requires an intact tertiary structure and is shared by the distantly related Netrin-1_NTR_. In contrast, the Sfrp1_CRD _does not mirror the effects of Sfrp1 over-expression but interacts *in vitro *with Fz receptors and antagonizes Wnt8-mediated β-catenin transcriptional activity, indicating that Wnt signalling modulation may involve multiple and differential interactions among Wnt, Fz and SFRPs.

These are somewhat surprising observations because it is generally accepted that Wnt-SFRP interaction takes place through the CRD domain due to its high degree of conservation with the extracellular portion of the Fz receptors [8,9]. Several studies in fact have provided convincing evidence that, when used in large amounts compared to Wnt protein concentration, SFRPs or their respective Sfrp_CRD _can efficiently block Wnt signalling in different contexts, such as in *Xenopus *axis formation [1,9], neural tube [26], somites [27] and heart formation [28], although a certain specificity among SFRPs has been observed. Furthermore, studies using cell lysates from co-transfected cell lines have shown physical interactions between Wnt1 or Wnt2 and Sfrp3_CRD _[8,9].

In contrast with this view, we have provided evidence in favour of the relevance of the NTR domain in SFRP-Wnt interaction. Although our data suggest that Sfrp_CRD _more likely interacts with Fz receptors, there are several possibilities worth considering as to why we may have failed to observe a clear interaction between Sfrp1_CRD _and Wnt. In the simpler scenario, the difference we have observed between the Sfrp1_NTR _and Sfrp1_CRD _domains' abilities to mimic the effect of the entire molecule could have been related to a differential translation efficiency of their respective mRNA within the embryos. However, this possibility seems quite unlikely because western blot analysis of embryo lysates injected with equimolar amounts of tagged molecules indicated that the different peptides were produced with similar efficiency and, if any, the Sfrp1_CRD _was expressed at higher levels. Similarly, Sfrp1_CRD-myc _was retrieved at consistently higher levels in the culture medium from transfected cell lines [29] and in primary cultures from retinal cells (unpublished observations). Furthermore, the reduction of the eye field observed after *Sfrp1*_*CRD *_injections was observed even with low mRNA doses.

A second possibility may relate to the stoichiometry of the Sfrp_CRD_-Wnt interaction. It has been proposed that a dimer of the CRD Fz8 domain binds Wnt8 [30] and dimerisation of the receptor may increase efficiency of signal transduction [31]. If Sfrp1_CRD _dimers form and bind Wnt8 more efficiently, it is possible that we may have missed this interaction since we noticed that we mostly immunoprecipitate the monomeric form (Figure 8a(iv)). This possibility, however, does not explain why in the reverse inmunoprecipitations (Additional file 2) the Wnt8-Sfrp1_CRD _immunocomplex was not observed. Similarly, it does not explain why Sfrp1_CRD _cannot counteract Wnt1/5/8 function *in vivo*, where both monomers and possible dimers seem to be present in similar amounts (Figure 3).

As a third possibility, failure of the Sfrp_CRD _to antagonize Wnt signalling may reflect specificity of binding. Although we have shown that Sfrp_CRD _failed to interact with Wnt8 and did not counteract the effect of Wnt1, Wnt5 and Wnt8 overexpression, we cannot exclude that Sfrp1 might show selectivity of binding through the two domains with Wnts other than those we have tested.

In agreement with our view of the importance of the Sfrp_NTR _domain in Wnt activity, several studies have provided indirect evidence in favour of the relevance of this domain. In *Drosophila*, the CRD motif of Dfz or Dfz2 is dispensable for Wg signal transduction and Frizzled proteins lacking the CRD can fully rescue the simultaneous loss of different Fz receptors or partially rescue the canonical signalling in *fz/fz2 *double mutants [32]. Furthermore, a carrier function for the CRD has been suggested in studies where the CRD domain of the *Drosophila *fz receptor has been substituted with the structurally distinct Wnt-binding domain or with wingless itself [33]. A recent study, aimed at demonstrating the interaction between Norrin and Fz4, failed to reveal a positive interaction between the CRD domain of all human SFRP family members and Xwnt8, which instead interacts with the CRD domain of Fz4, 5, 7 and 8 (see Figure 2 in [34]). Furthermore, *in vitro *analysis of the interaction between Sfrp1 and Wingless mapped the relevant SFRP binding site to the carboxyl terminus of the protein [4]. Our biochemical and functional data are in line with this set of data, strongly supporting the proposal that the NTR domain has a relevant role in mediating Sfrp function. This role is conserved also in distantly related domains. Indeed, the NTR of Sfrp1, 2, and 5 shares a quite similar pattern of cysteine spacing, related to that of Netrin-1. Conformational similarities are, therefore, likely to explain why over-expression of Sfrp1_NTR_, Sfrp2_NTR _and Netrin-1_NTR _results in all cases in forebrain expansion and effective inhibition of Wnt8-induced β-catenin activation. In contrast, Sfrp3_NTR_and Sfrp4_NTR _display a different cysteine spacing and, thus, a distinct pattern of disulphide bonds [5], supporting that variations in the NTR structural features could underlie the differences in activities observed among the distinct subgroups of the family [5,16], as we have observed with Sfrp3_NTR_.

The crystallographic resolution of the structure of the mouse Sfrp3 and Fz8 CRD domains revealed the potential for the different CRDs to homo- or heterodimerise [10]. This potential has also been demonstrated in biochemical studies where SFRPs and Fzs and/or their CRDs have been shown to form homo- and/or hetero-complexes [8,24,31]. In line with these data, we have demonstrated a physical interaction between Sfrp1_CRD _and Fz5 and Fz2. This binding may very well justify the potential of the Sfrp1_CRD _to antagonize, albeit with lower efficiency, Wnt8-induced β-catenin activation, as we have observed in our experimental conditions mimicking the physiological extracellular interactions among Fz, Wnt and SFRPs. This interaction also provides a mechanism, based on functional inactivation of the receptor, to explain why, in many studies, addition of high levels of the CRD alone is sufficient to prevent Wnt signalling activation. The reason why, in our studies, *Sfrp1*_*CRD *_over-expression in medaka fish embryos seems to synergize rather than prevent the effect of Wnt8 over-expression (Figure 2) is, however, unclear. As a tempting speculation, Sfrp1_CRD _may have higher affinity for Fz receptors that, like Fz2 [35], are involved in mediating non-canonical signalling, which, in turn, has been shown to antagonize the Wnt canonical pathway during eye field specification [36]. Alternatively, in the embryo, Sfrp1_CRD _may interfere with other cell signalling pathways, as demonstrated for the CRD of Sizzled, a related family member that binds and inhibits Tolloid/BMP1, metalloproteases that normally degrade the BMP inhibitor chordin, thereby promoting BMP signalling [23,37].

## Conclusion

**Figure 9:**
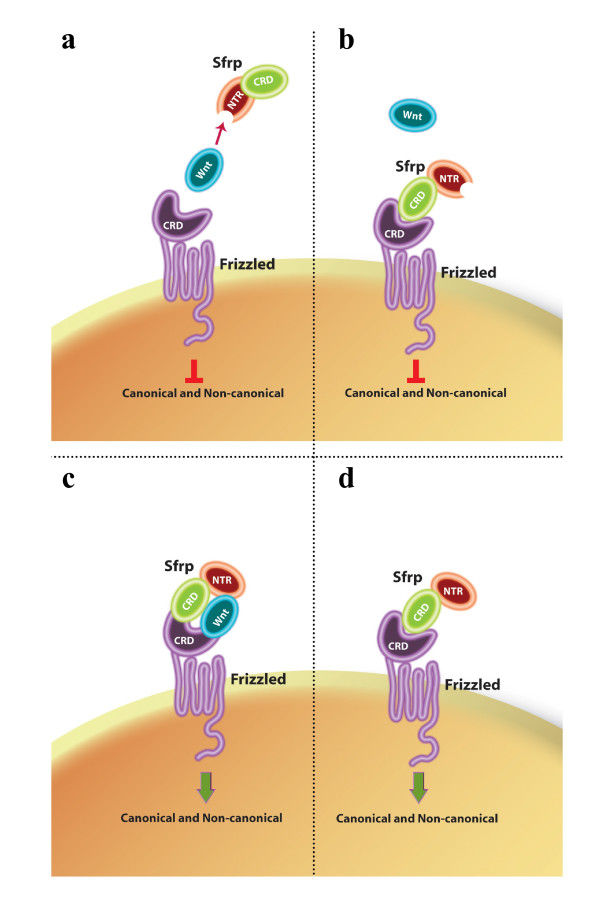
**SFRP mode of action may rely on multiple interactions with Wnt ligands and/or Frizzled receptors**. Schematic representation of possible mechanisms by which SFRPs could modulate Wnt/Frizzled signalling. **(a) **SFRPs can antagonize Wnt activity by directly binding to the ligand through its Netrin-related domain. **(b) **SFRPs could interact directly with Frizzled receptors through their corresponding CRD motifs and prevent signal transduction. **(c) **Frizzled, Wnt and SFRP molecules could form heterotrimeric complexes, where SFRP could present the Wnt ligand to the Frizzled receptor thanks to the differential interactions of the CRD and NTR domains. **(d) **In the absence of Wnt ligands, SFRPs can directly bind a Frizzled receptor and transduce a signal. See the text for further details.

We have provided functional and biochemical evidence that the NTR, but not the CRD, domain of Sfrp1 mimics the function of the entire molecule. These results challenge several reports implying that the CRD domain of SFRPs, due to its homology with the proposed Wnt binding region on Fz receptors, interferes with Wnt signalling by binding and sequestering the ligand [8,9]. These apparent contradictions can, however, be reconciled with two assumptions. First, SFRPs of different subgroups have different biochemical interactions with Wnt ligands. In support of this assumption, plasmon resonance binding studies using Sfrp1, 2, 3, 4 and Wnt3a and Wnt5 have shown that Wnt5 binds preferentially to Sfrp1 and 2, while Wnt3a binds at least two sites in Sfrp1, 2, 4 and one in Sfrp3 [16]. Second, SFRP molecules interact with both Wnt and Fz in multiple ways and these interactions can modulate signal transduction in either a positive or negative manner. In this view, there are several possible mechanisms by which SFRPs can modulate Wnt signalling (Figure 9). SFRP could sequester Wnt ligands through the NTR domain, thus acting as antagonists (Figure 9a; this study) or act in a dominant-negative manner through the formation of inactive complexes with Fz receptors, preventing signal activation (Figure 9b; as proposed previously [8], and this study). Alternatively, SFRPs could favour Wnt-Fz interaction by simultaneously binding to both molecules and, thus, synergizing with signal activation (Figure 9c), as reported previously [4]. Finally, in the absence of Wnt ligands, Sfrp_CRD_-Fz_CRD _heterodimer formation could trigger signal transduction (Figure 9d), as proposed previously [24]. Notably, the activation of the Fz receptors by a proposed ligand-antagonist is not unique to SFRP1, as Dickkopf2, which belongs to a different family of Wnt antagonists, can activate Wnt canonical signalling cooperating with at least three different Fzs [38].

Genetic manipulations selectively eliminating one or the other domain of SFRPs may provide further insights and help resolve the accuracy of these models. Additional studies characterizing the functionally relevant interactions among Sfrp_NTR_-Wnt or Sfrp_CRD_-Fz pairs are also undoubtedly needed. Interaction with additional components of the Wnt signalling cascade also needs to be addressed. Particularly relevant might be the contributions of proteoglycans, which are known to bind Wnts [39] and may additionally interact with the Sfrp_NTR _(PE, unpublished observations). An accurate establishment of SFRP mode of action is indeed particularly important given the growing interest in these molecules raised by the observations that their expression is altered in different type of cancers, bone pathologies, retinal degenerations and hypophosphatemic diseases, pointing to their potential value as therapeutic targets.

## Materials and methods

### Whole-mount *in situ *hybridisation

Whole-mount *in situ *hybridizations were performed in medaka embryos using digoxigenin- and fluorescein-labelled riboprobes. A minimum of 40 embryos were hybridized for each marker and condition. All embryos shown correspond to Iwamatsu stage 19–20 [40].

### Construct generation

*olSfrp1*, *mWnt8a*, *zWnt5 and zWnt1 *expression constructs have been described [13,36,41,42]. z*Sizzled *was a kind gift of Dr Hibi and x*Sizzled *of Dr E De Robertis. Medaka *Sfrp2 *full length clone corresponds to the expressed sequence tag MF01SSA080C03, kindly provided by Dr. Takeda. *zSfrp3 *and *olNetrin-1 *where cloned by RT-PCR using specific primers. Full length, truncated and chimerical coding sequences of *Sfrp1*, *Sfrp2*, *Sfrp3 *and *Netrin-1 *where cloned by PCR into pCS2+. All chimerical constructs where designed so that the signal peptide of the corresponding protein was fused in frame with the linker region that precedes the NTR domain, ensuring proper secretion of the corresponding peptide (Figure 1). Cysteine to serine mutations were introduced into the NTR of both Sfrp1 and Netrin-1 by PCR. Given the structural similarity between serine and cysteine, this substitution is expected to disrupt di-sulphide bridge formation without altering the secondary structure of the peptide. Carboxy-terminal 3xHA tagged constructs of Sfrp1, Sfrp1_CRD _and Sfrp1_NTR _were generated with linker oligos. All constructs were fully sequenced to ensure in-frame fusions.

### mRNA and morpholino injections

pCS2 plasmids were linearised and transcribed *in vitro *using the SP6 Message mMachine kit (Ambion, Austin, TX, USA). The synthesized mRNA was purified and injected into two-cell stage embryos at different concentrations (titration curve: 50–300 ng/μl) and the severity of the induced phenotypes was dose dependent in all the cases. Injection solutions included 30 ng/ml of hGFP mRNA as a lineage tracer. Selected working concentrations correspond to equimolecular amounts of the different *Sfrp *mRNAs (full length, truncated and chimerical) to obtain equivalent protein levels (Tables 1 and 2). Mo studies were performed as previously described [13] using the following tested Mo (Gene Tools, LLC, Philomath, OR, USA) designed against *olSfrp1*: 5'-CTGTGTTT GTAGGAACCTCGACTGG-3'. Mo were injected at the final concentration of 0.3 mM into one blastomere of embryos at the two-cell stage. For co-injection experiments, 60 ng of *Sfrp1 *or 30 ng of *Sfrp1*_*CRD *_or 35 ng of *Sfrp1*_*NTR *_mRNAs were used. At least three independent experiments were conducted for each marker and condition.

### Protein expression and immunoprecipitations

To determine the efficiency of translation of the Sfrp1 and its derivatives, triply-HA tagged constructs were generated (see above) and their respective mRNAs were injected into medaka embryos in equimolecular amounts (*Sfrp1-3HA*, 200 ng/μl; *Sfrp1*_*CRD*_-*3HA*, 100 ng/μl; and *Sfrp1*_*NTR*_-*3HA*, 120 ng/μl) together with *GFP *mRNA as a tracer. For each construct, 30 embryos were treated with lysis buffer (150 mM NaCl; 1% NP40; 50 mM Tris pH 8; 10 μg/ml aprotinin; 10 μg/ml leupeptin and 1 mM phenylmethanesulphonylfluoride (PMSF). Lysates were precipitated with a polyclonal anti-HA (Sigma-Aldrich, St Louis, MI, USA) and Protein G-Sepharose for enrichment. The protein complex present in each of the pellets was re-suspended in 2 × SDS sample buffer containing 1 M urea. The proteins were resolved by SDS-PAGE blotted and the membranes probed with a monoclonal anti-HA (Sigma-Aldrich). Proteins from total cell extracts were subjected to SDS-PAGE, blotted and the membranes probed with an anti-GFP antibody (Molecular Probes, Invitrogen, Carlsbad CA, USA) and a secondary anti-rabbit-POD antibody.

Sub-confluent HEK 293T cells were transiently and separately transfected with constructs encoding chick Wnt8c-HA, chick Sfrp1-myc or Sfrp1_CRD-myc _or Sfrp1_NTR-myc _in 2% fetal calf serum. After 2 days, the conditioned media were collected and clarified by centrifugation. The amount of protein present in the conditioned media was evaluated by western blot and similar amounts of peptides derived from each Sfrp1-myc present in the conditioned media were mixed with conditioned medium from Wnt8-HA or mock transfected for 2 hours. Sample volumes were adjusted to 600 μl with lysis buffer (as above). Proteins from conditioned media were precipitated with 3 μg of an anti-HA polyclonal antibody (Sigma-Aldrich) and Protein G-Sepharose. After four washes with lysis buffer, the protein complex was subjected to SDS-PAGE, blotted and the membranes probed with a monoclonal anti-myc antibody (9E10) and a secondary anti-mouse-POD antibody. Signal was detected with the Advanced ECL Western blotting detection Kit analysis (GE Healthcare Life Sciences, Pollards Wood, Buckinghamshire, UK). Reverse inmunoprecipitation experiments were performed using similar incubations of conditioned media. Proteins were precipitated with a polyclonal anti-myc antibody (SIGMA). The immunocomplexes were subjected to SDS-PAGE, blotted and the membranes probed with a monoclonal anti-myc antibody (9E10) and a secondary anti-mouse-POD antibody.

For Fz2 and Fz5 immunoprecipitations, HEK 293T cells were transiently transfected with mouse *Fz2-HA*, chick-*Sfrp1-myc *or *Sfrp1*_*CRD*-*myc *_or *Sfrp1*_*NTR*-*myc *_or cotransfected with mouse *Fz5-HA *and chick-*Sfrp1-myc *or *Sfrp1*_*CRD*-*myc *_or *Sfrp1*_*NTR*-*myc *_expression constructs. After 2 days, cells were scraped in lysis buffer (as above). Immunoprecipitations were performed as previously described [24].

### Reporter assays

Dissociated cells from embryonic day (E)5 central retinas were prepared as described [29], seeded in 24-well plates and transfected 3 hours later using the FuGENE HD Transfection Reagent (Roche, Nutley, NJ, USA). In each case the 700 ng/well of total DNA contained 200 ng of a plasmid containing a 4xLef-1 responsive luciferase reporter and 50 ng of pRL-TK (Promega, Madison, WI, USA) together with variable amounts of the effector plasmids or the empty vector. After 24 hours, luciferase activities were determined using a dual-luciferase assay system (Promega). The LEF-1 reporter luciferase activity was normalized with that of the *Renilla *luciferase to account for transfection efficiency. Data were statistically evaluated using the SPSS v15.0 software (SPSS Inc., Chicago, Illinois, USA) applying a one-way ANOVA test plus *post hoc *test (Dunnet test).

### Image acquisition

Live embryos were visualized at room temperature under a Leica stereomicroscope equipped with a PLANAPO objective. Embryos processed for *in situ *hybridization were fixed with 4% paraformaldehyde (PFA) and equilibrated in 80% glycerol. After removal of the yolk, embryos were mounted and visualized under a Leica microscope. In all cases, images were captured with a Leica digital camera controlled by the Leica software.

## Abbreviations

CRD: Cysteine-rich domain; E: Embryonic day; Fz: Frizzled; HA: Haemagglutinin; Mo: Morpholino; NTR: Netrin-related motif; SFRP: Secreted frizzled related protein; BMP: Bone morphogenetic protein: PMSF: Phenylmethylsulphonyl fluoride.

## Competing interests

The authors declare that they have no competing interests.

## Authors' contributions

JLR, PE and PB conceived, designed and discussed the study. JLR constructed most of the plasmids and performed and analysed the overexpression studies. PE constructed part of the plasmids, performed the reporter assays and immnoprecipitation studies and participated in the analysis of *in vivo *studies. JMR performed and analysed the Mo-rescue studies as well as Wnt1/Sfrp1 co-injection studies. JLR and PE wrote a draft of the manuscript. PB wrote the final version of the manuscript, which was approved by all authors.

## Supplementary Material

Additional File 1 SFRP_CRD _peptides cannot rescue the *Wnt8*- or *Wnt5*-induced over-expression phenotype. All the panels are dorsal views of embryos at stage 19–20 (optic vesicle stage) injected with *GFP *mRNA alone or combined with *Wnt8*, *Wnt5*, *Sfrp1*_*CRD*-2_, or *Sfrp3*_*CRD *_mRNA as indicated. Note that *Sfrp1*_*CRD*-2 _(b) behaves as *Sfrp1*_*CRD *_(Figure 1c) in over-expression assays, while *Sfrp3*_*CRD *_has no evident effect even at high concentrations (c; 300 ng/μl). Consistently, neither *Sfrp1*_*CRD *_nor *Sfrp3*_*CRD *_can rescue the phenotype induced upon *Wnt8 *(_*D*-*F*_) or *Wnt5 *(_*G*-*I*_) over-expression. See Tables 1 and 2 for details. Scale bar: 0.1 mm. Click here for file

Additional File 2 Wnt8 binds to Sfrp1 and Sfrp1_NTR _while Sfrp1_CRD _binds to Frizzled 2. **(a) **Mixed conditioned media used in Figure 8a (see legend) were precipitated with a polyclonal anti-myc and blotted with a monoclonal anti-HA (upper panel). Controls for inputs (middle and lower panels) are the same as those described in Figure 8a(ii and iii). Wnt8-HA co-immunoprecipitated with both Sfrp1-myc and Sfrp1_NTR-myc _while Sfrp1_CRD-myc _did not. **(b) **HEK 293T cells were transiently co-transfected with *Fz2-HA *constructs together with *Sfrp1*-_*myc*_, *Sfrp1*_*CRD*-*myc *_or *Sfrp1*_NTR-*myc*_. Proteins from cell lysates were precipitated with anti-HA and then blotted with anti-myc antibody. Note that Sfrp1 and Sfrp1_CRD _(red asterisks) interact with Fz2 while the Sfrp1_NTR _does not. **(c) **Conditioned media from mock transfected cells were mixed with Sfrp1-myc, Sfrp1_NTR-myc _or Sfrp1_CRD-myc _conditioned media (as above). Addition of anti-HA polyclonal antibodies did not cause unspecific immunoprecipitations as revealed by western blotting with anti-Myc monoclonal antibody. **(d) **Addition of anti-HA polyclonal antibodies did not cause unspecific immunoprecipitations in cell lysates from mock and Sfrp1-myc, Sfrp1_NTR-myc _or Sfrp1_CRD-myc _co-transfected cells as revealed by western blots with anti-Myc antibody. Click here for file

Additional File 3 Wnt8/Fz5 mediated activation of β-catenin transcriptional activity in dissociated embryonic retinal cells is inhibited by soluble Sfrp1 and Sfrp1_NTR _as well as by the Sfrp1_CRD_. **(a) **E5 embryonic chick retinal cells were dissociated and co-transfected with a reporter plasmid containing 4xLef-1 responsive element, the control plasmid pRLTK and the effector plasmids for each condition. In retinal cells, endogenous β-catenin transcriptional activity is low and barely modified by transfection of *Fz5 *alone or by the co-transfection of *Fz5 *with *Sfrp1*, *Sfrp1*_*CRD *_or *Sfrp1*_*NTR*_. In contrast, strong reporter activation is observed upon *Fz5 *and *Wnt8 *co-transfection. **(b) **HEK 293T cells grown in 2% fetal calf serum were transfected with *Sfrp1-myc*, *Sfrp1*_*CRD*-*myc*_, or *Sfrp1*_*NTR*-*myc*_. Two days later the conditioned media were collected and similar amounts of proteins were added to dissociated retinal cell cultures co-transfected with a reporter plasmid (as above), *pRLTK*, *Wnt8 *and *Fz5*. TCF-luciferase activity was measured after 24 hours of incubation. Note how the conditioned media strongly inhibit reporter activities. Data represent means ± standard error from three separate experiments performed in triplicates. **(c) **Cells dissociated from E5 embryonic retinas were co-transfected with a reporter plasmid containing 4xLef-1 responsive element together with *Wnt8*, *Fz5 *(100 ng) in combination with the PCDNA plasmid alone (200 ng) or containing *Sfrp1*, *zSizzled*, *Sfrp1*_*CRD *_or *zSizzled*_*CRD *_as indicated in the graph. *Wnt8*/*Fz5 *co-transfection activated the reporter expression. This activation was significantly inhibited by the addition of Sfrp1 and Sfrp1_CRD _while with less efficiency by the addition of *zSizzled *or *zSizzled*_*CRD*_. Similar results were obtained with the *Xenopus *sizzled constructs. *p < 0.05; **p < 0.01; ***p < 0.001). Click here for file
