# Supplementary figures and images for: The Netrin-related domain of Sfrp1 interacts with Wnt ligands and antagonizes their activity in the anterior neural plate
Source: Neural Dev. 2008 Aug 20;3:19. doi: 10.1186/1749-8104-3-19 (PMC2542364; doi:10.1186/1749-8104-3-19)

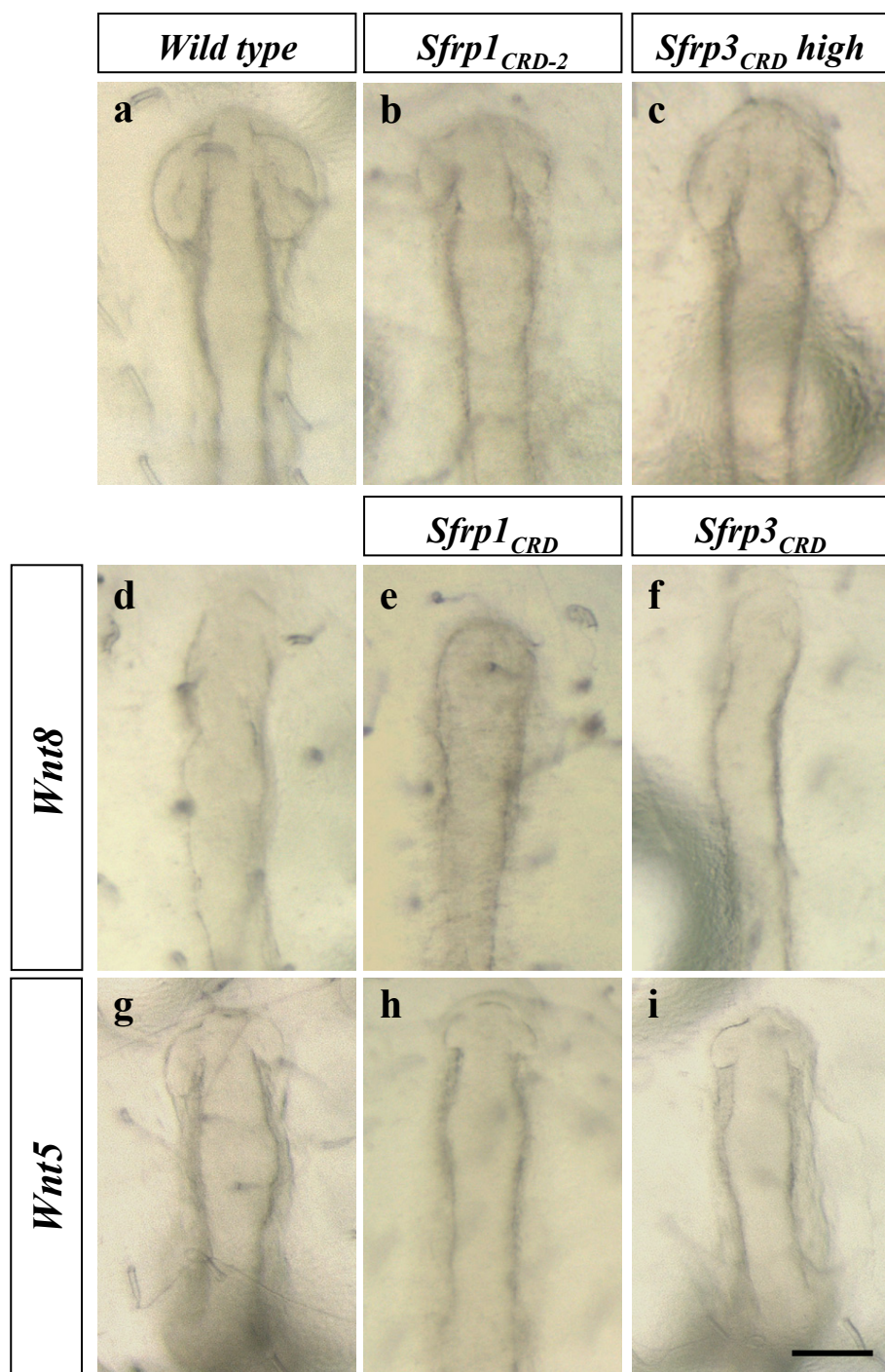

**Figure S1**

Supplement: Additional File 1 — SFRPCRD peptides cannot rescue the Wnt8- or Wnt5-induced over-expression phenotype. All the panels are dorsal views of embryos at stage 19–20 (optic vesicle stage) injected with GFP mRNA alone or combined with Wnt8, Wnt5, Sfrp1CRD-2, or Sfrp3CRD mRNA as indicated. Note that Sfrp1CRD-2 (b) behaves as Sfrp1CRD (Figure 1c) in over-expression assays, while Sfrp3CRD has no evident effect even at high concentrations (c; 300 ng/μl). Consistently, neither Sfrp1CRD nor Sfrp3CRD can rescue the phenotype induced upon Wnt8 (D-F) or Wnt5 (G-I) over-expression. See Tables 1 and 2 for details. Scale bar: 0.1 mm. [file 1749-8104-3-19-S1.pdf]

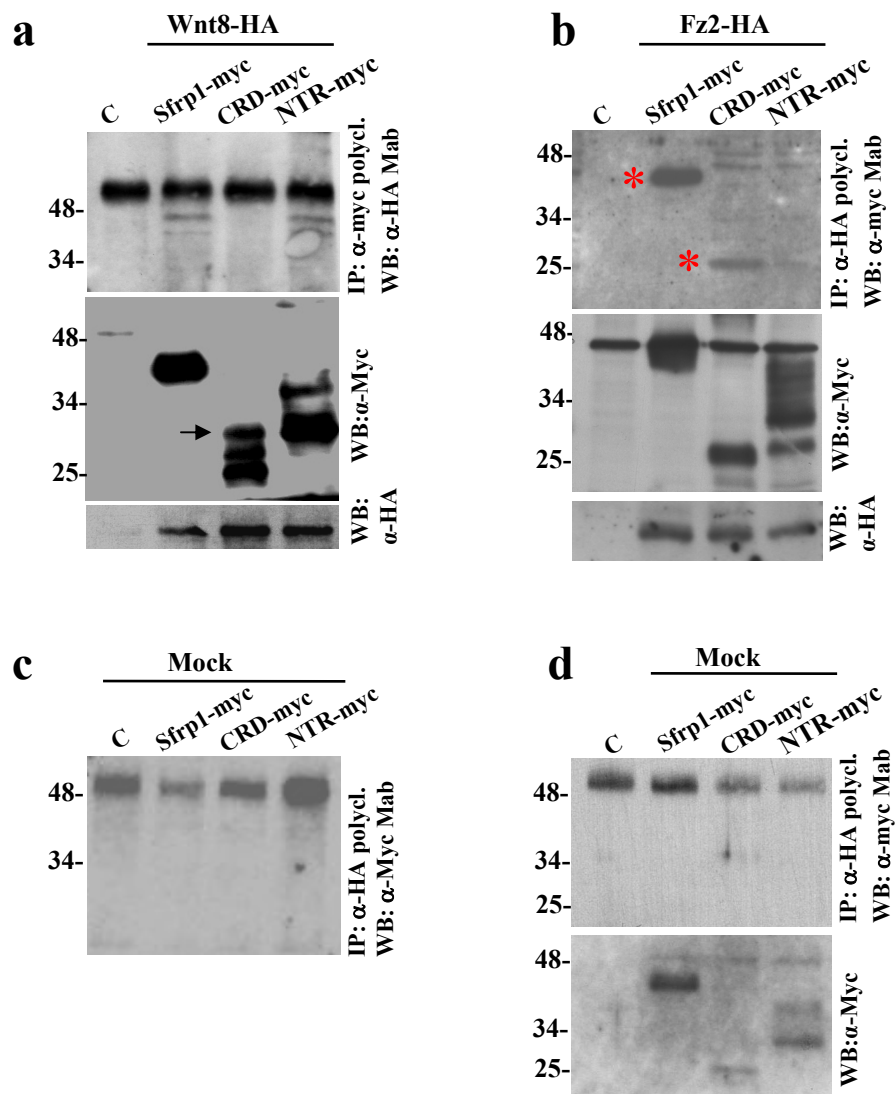

**Figure S2**

Supplement: Additional File 2 — Wnt8 binds to Sfrp1 and Sfrp1NTR while Sfrp1CRD binds to Frizzled 2. (a) Mixed conditioned media used in Figure 8a (see legend) were precipitated with a polyclonal anti-myc and blotted with a monoclonal anti-HA (upper panel). Controls for inputs (middle and lower panels) are the same as those described in Figure 8a(ii and iii). Wnt8-HA co-immunoprecipitated with both Sfrp1-myc and Sfrp1NTR-myc while Sfrp1CRD-myc did not. (b) HEK 293T cells were transiently co-transfected with Fz2-HA constructs together with Sfrp1-myc, Sfrp1CRD-myc or Sfrp1NTR-myc. Proteins from cell lysates were precipitated with anti-HA and then blotted with anti-myc antibody. Note that Sfrp1 and Sfrp1CRD (red asterisks) interact with Fz2 while the Sfrp1NTR does not. (c) Conditioned media from mock transfected cells were mixed with Sfrp1-myc, Sfrp1NTR-myc or Sfrp1CRD-myc conditioned media (as above). Addition of anti-HA polyclonal antibodies did not cause unspecific immunoprecipitations as revealed by western blotting with anti-Myc monoclonal antibody. (d) Addition of anti-HA polyclonal antibodies did not cause unspecific immunoprecipitations in cell lysates from mock and Sfrp1-myc, Sfrp1NTR-myc or Sfrp1CRD-myc co-transfected cells as revealed by western blots with anti-Myc antibody. [file 1749-8104-3-19-S2.pdf]

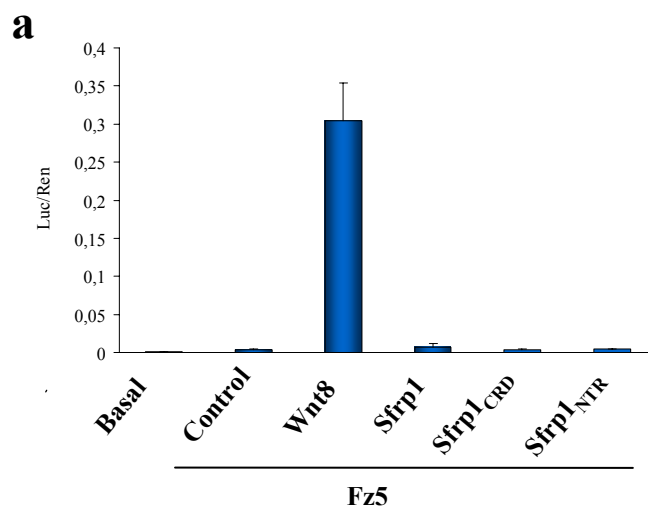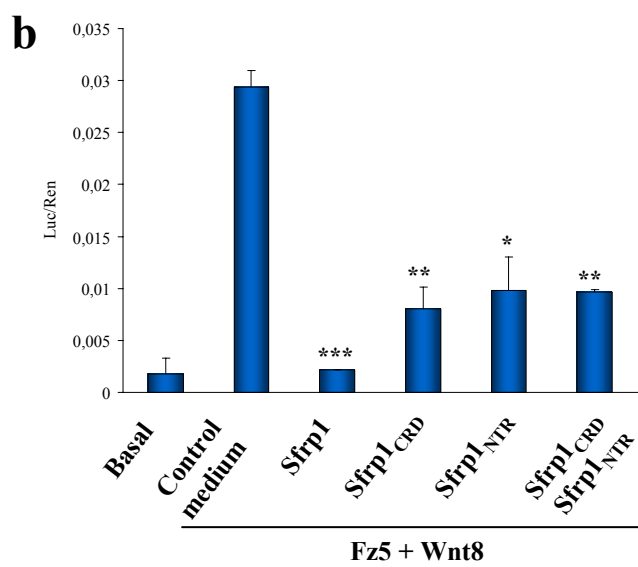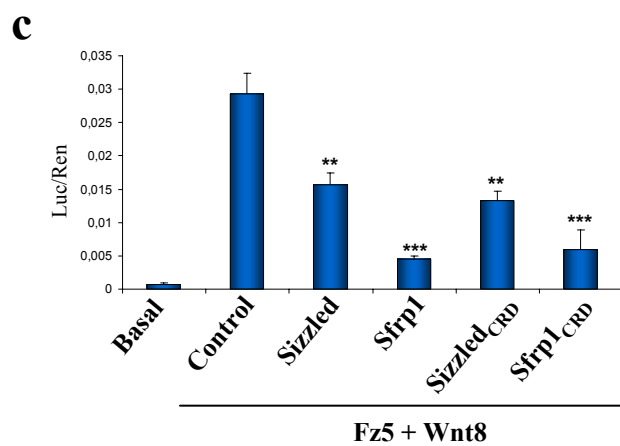

**Figure S3**

Supplement: Additional File 3 — Wnt8/Fz5 mediated activation of β-catenin transcriptional activity in dissociated embryonic retinal cells is inhibited by soluble Sfrp1 and Sfrp1NTR as well as by the Sfrp1CRD. (a) E5 embryonic chick retinal cells were dissociated and co-transfected with a reporter plasmid containing 4xLef-1 responsive element, the control plasmid pRLTK and the effector plasmids for each condition. In retinal cells, endogenous β-catenin transcriptional activity is low and barely modified by transfection of Fz5 alone or by the co-transfection of Fz5 with Sfrp1, Sfrp1CRD or Sfrp1NTR. In contrast, strong reporter activation is observed upon Fz5 and Wnt8 co-transfection. (b) HEK 293T cells grown in 2% fetal calf serum were transfected with Sfrp1-myc, Sfrp1CRD-myc, or Sfrp1NTR-myc. Two days later the conditioned media were collected and similar amounts of proteins were added to dissociated retinal cell cultures co-transfected with a reporter plasmid (as above), pRLTK, Wnt8 and Fz5. TCF-luciferase activity was measured after 24 hours of incubation. Note how the conditioned media strongly inhibit reporter activities. Data represent means ± standard error from three separate experiments performed in triplicates. (c) Cells dissociated from E5 embryonic retinas were co-transfected with a reporter plasmid containing 4xLef-1 responsive element together with Wnt8, Fz5 (100 ng) in combination with the PCDNA plasmid alone (200 ng) or containing Sfrp1, zSizzled, Sfrp1CRD or zSizzledCRD as indicated in the graph. Wnt8/Fz5 co-transfection activated the reporter expression. This activation was significantly inhibited by the addition of Sfrp1 and Sfrp1CRD while with less efficiency by the addition of zSizzled or zSizzledCRD. Similar results were obtained with the Xenopus sizzled constructs. *p < 0.05; **p < 0.01; ***p < 0.001). [file 1749-8104-3-19-S3.pdf]
